# Imatinib therapy of chronic myeloid leukemia significantly reduces carnitine cell intake, resulting in adverse events

**DOI:** 10.1016/j.molmet.2024.102016

**Published:** 2024-08-23

**Authors:** Pavel Burda, Alzbeta Hlavackova, Vendula Polivkova, Nikola Curik, Adam Laznicka, Jitka Krizkova, Jiri Suttnar, Pavel Klener, Katerina Machova Polakova

**Affiliations:** 1 Institute of Hematology and Blood Transfusion, Prague, Czech Republic; 2 Institute of Pathological Physiology, First Faculty of Medicine, Charles University, Prague, Czech Republic; 3 First Medical Department- Dept. of Hematology, First Faculty of Medicine and General University Hospital, Charles University, Prague, Czech Republic

**Keywords:** Imatinib, Carnitine, OCTN2, CML, TKI therapy side effects

## Abstract

**Objective:**

A prominent, safe and efficient therapy for patients with chronic myeloid leukemia (CML) is inhibiting oncogenic protein BCR::ABL1 in a targeted manner with imatinib, a tyrosine kinase inhibitor. A substantial part of patients treated with imatinib report skeletomuscular adverse events affecting their quality of life. OCTN2 membrane transporter is involved in imatinib transportation into the cells. At the same time, the crucial physiological role of OCTN2 is cellular uptake of carnitine which is an essential co-factor for the mitochondrial β-oxidation pathway. This work investigates the impact of imatinib treatment on carnitine intake and energy metabolism of muscle cells.

**Methods:**

HTB-153 (human rhabdomyosarcoma) cell line and KCL-22 (CML cell line) were used to study the impact of imatinib treatment on intracellular levels of carnitine and vice versa. The energy metabolism changes in cells treated by imatinib were quantified and compared to changes in cells exposed to highly specific OCTN2 inhibitor vinorelbine. Mouse models were used to test whether *in vitro* observations are also achieved *in vivo* in thigh muscle tissue. The analytes of interest were quantified using a Prominence HPLC system coupled with a tandem mass spectrometer.

**Results:**

This work showed that through the carnitine-specific transporter OCTN2, imatinib and carnitine intake competed unequally and intracellular carnitine concentrations were significantly reduced. In contrast, carnitine preincubation did not influence imatinib cell intake or interfere with leukemia cell targeting. Blocking the intracellular supply of carnitine with imatinib significantly reduced the production of most Krebs cycle metabolites and ATP. However, subsequent carnitine supplementation rescued mitochondrial energy production. Due to specific inhibition of OCTN2 activity, the influx of carnitine was blocked and mitochondrial energy metabolism was impaired in muscle cells *in vitro* and in thigh muscle tissue in a mouse model.

**Conclusions:**

This preclinical experimental study revealed detrimental effect of imatinib on carnitine-mediated energy metabolism of muscle cells providing a possible molecular background of the frequently occurred side effects during imatinib therapy such as fatigue, muscle pain and cramps.

## Highlights

• Imatinib therapy of chronic myeloid leukemia outcompetes carnitine for OCTN2 cell intake.
• Reduced carnitine cell intake impairs energy metabolism and harming muscle tissues.
• Carnitine supplementation doesn't affect imatinib's influx or BCR::ABL1 inhibition.
• High-dose carnitine rescued impaired mitochondrial energy metabolism after imatinib treatment.

## Graphical abstract

**Figure:**
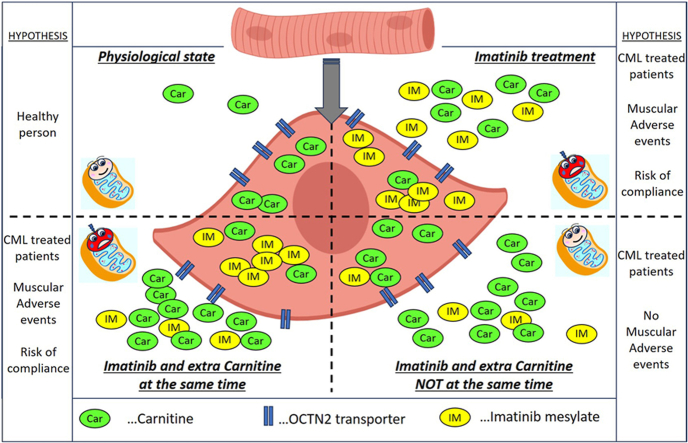

## 1 Introduction

Targeted inhibition of oncogenic protein BCR::ABL1 with tyrosine kinase inhibitors (TKIs) is a front-line therapy in patients with chronic myeloid leukemia (CML) and Ph + acute lymphoblastic leukemia. Imatinib is the most frequently applied TKI as a first-line treatment against CML because of its safety, efficacy and price (generics). Nevertheless, up to 80% of CML patients treated with imatinib report substantial side effects. Imatinib is also administered in patients with unresectable/metastatic gastrointestinal stromal tumors (GISTs) and inhibits the activity of KIT and PDGFR at the dose necessary for BCR::ABL1 inhibition [1]. Myalgia and other musculoskeletal reactions are among the most frequent adverse events on imatinib treatment [2]. Muscle pain or cramps were self-reported in up to 90% of imatinib-treated CML patients in the first months, after therapy start followed by swelling in the hands, legs, feet, or around the eyes (75%) and fatigue (50%) [3], affecting quality of life (QoL) [4]. The most frequently reported adverse events of nilotinib are rash (31%), pruritus (15%), headache (14%), nausea and fatigue (11%). Myalgia and muscle spasms occurs in approximately 10% and 7% of nilotinib-treated patients, respectively [5]. The most common dasatinib-related non-hematologic adverse events are fluid retention (superficial edema and pleural effusion), which is reported in up to 25% of patients; myalgia, including muscle inflammation or musculoskeletal pain (22%); diarrhea (19%); and headache (13%) [6]. Most persistent non-hematological toxicities related to bosutinib include diarrhea (84% of patients), nausea (43%), vomiting (32%) and rashes (22%) [7]. Muscle pain and/or cramps are rarely reported for 3rd generation TKIs. Most serious ponatinib-related adverse event is cardiovascular toxicity which incidence is dose related [8]. Asciminib shows less toxicity than other TKIs, with reported nausea, headache, fatigue, joint pain, and high blood pressure (occurring in 20–30% patients) [9].

The bioavailability of imatinib and therapeutic window can be affected by the expression and activity of drug carriers that transport a drug through the plasmatic membrane of leukemic cells and cells in the gastrointestinal tract. Imatinib exhibited significantly higher affinity to the SLC group (solute carrier proteins) of transporters OCTN2 (*SLC22A5*), OATP1B3 (*SLC21A6*) and OATP1A2 (*SLC21A3*) compared to others OCT1 (*SLC22A1*), OCT2 (*SLC22A2*), OCT3 (*SLC22A3*), OAT1 (*SLC22A6*), OAT2 (*SLC22A7*), OAT3 (*SLC22A8*) and OCTN1 (*SLC22A4*) [10]. Interestingly, OCTN2 transporter is essential transporter of the l-carnitine into cells. l-carnitine (and acetyl l-carnitine and propionyl l-carnitine) is either synthesized by the body (usually approximately 25% from total body reserve), the majority is obtained from dietary routes [11]; in addition l-carnitine (further referred as carnitine) is intrinsically involved in mitochondrial metabolism and function, as it plays a key role in fatty acid beta-oxidation and energy metabolism. Carnitine binds acyl residues derived from the intermediary metabolism of fatty acids and functions as a scavenger to help eliminate these residues (as previously reviewed by [12]). In addition to transporting free fatty acids across the inner mitochondrial membrane, carnitine modulates their oxidation rate and is involved in the regulation of vital cellular functions. The carnitine transporter OCTN2 is highly expressed in muscles, heart, and kidney regions and on the surface of hematopoietic cells; in addition, OCTN2 operates sodium-dependent transport of carnitine and sodium-independent organic cation transport. Impaired carnitine transport through OCTN2 results in urinary carnitine waste, low serum carnitine levels (0–8 μM, normal 25–50 μM), and decreased intracellular carnitine accumulation [13,14]. Carnitine and its derivatives show promising effects in the treatment of chronic conditions and diseases associated with mitochondrial dysfunction. In a prior study, carnitine was examined in patients with invasive malignancy and fatigue, and carnitine deficiency was reported in more than 30% of cancer patients at baseline [15]. In addition, several studies have demonstrated that carnitine supplementation relieves muscle cramps in patients with liver cirrhosis and hemodialyzed patients [16,17].

Among the TKIs approved for CML treatment in the USA and EU—imatinib, dasatinib, nilotinib, bosutinib, ponatinib, and asciminib—imatinib is the only one whose absorption depends on both influx (e.g., OCTN2, OATP1A2, OCT1) and efflux (e.g., ABCB1, ABCG2) transporters. The other TKIs rely solely on efflux transporters, with their cellular intake being primarily a passive process (reviewed in [18]). Consequently, these TKIs are not expected to interfere with transport via OCTN2. This work demonstrated that the transport molecule OCTN2, a dominant transporter for cellular intake of carnitine, is competitively and preferentially used for imatinib intake. The unequal competition for OCTN2 between imatinib and carnitine led to a reduction or deficiency of intracellular carnitine; this effect was associated with a limited rate of metabolite production in the TCA (Krebs) cycle, diminished production of lactate and depletion of energy-rich molecules. The impaired energy metabolism was rescued by high-dose carnitine supplementation followed imatinib treatment.

## 2 Materials and methods

### 2.1 Cell lines and cell culture

The human rhabdomyosarcoma cell line HTB-153 (adherent cells, HS 729, ATCC, Manassas, VA, USA) and the human leukemic cell line KCL-22 (suspension cells, ACC 519, DSMZ, Braunschweig, Germany) were used for *in vitro* experiments. We selected *BCR::ABL1*-positive cell line KCL-22 as a representative CML and HTB-153 as a rhabdomyosarcoma cell line biologically resembling the muscular tissue. Both cell lines are commercially available and well-characterized. Importantly, no aberrations on locus 5q31.1, where the *SLC22A5* gene is located, were detected in these cells.

The cell lines were handled and cultivated in appropriate medium according to the recommendations of the supplier. The following doses of imatinib were used for incubation: in KCL-22, up to a sublethal dose (1 μM) was used for long-term cultivation, and higher concentrations (up to 3 μM) could be used in a 24-hour experiment without impairing cell vitality as measured by FACS (apoptosis rate). For HTB-153, 8 μM imatinib was equally distributed among cells growing in layers and was a nontoxic dose for times in experiments. Carnitine-isotopically labelled Car-D3 (Sigma–Aldrich, St. Louis, MO, USA) was used at concentrations up to 12 μM. Vinorelbine (VNR), an OCTN2-specific inhibitor (A10976, Adooq Bioscience LLC, CA, USA), was used at a concentration of 50 μg/ml.

### 2.2 Protein expression analysis

For immunoblotting analyses, 10^7^ cells were lysed with RIPA buffer and gently sonicated. The protein lysates (20 μg) were resolved in a 4–12% gradient Bis-Tris gel (NuPageLife Technologies, Carlsbad, CA, USA) and blotted. For western blot analyses, the following primary antibodies were used: anti-OCTN2 (Thermo Fisher Scientific, Waltham, MA, USA), anti-OCT1 (Abcam, Cambridge, UK), anti-OATP1A2 (Abcam), anti-OATP1B3 (Abcam), and anti-β-actin (Santa Cruz Biotechnology, Dallas, TX, USA).

### 2.3 Measurement of intracellular concentrations of imatinib, carnitine and metabolites

Intracellular concentrations of imatinib and carnitines were measured after extraction from cell pellets by 1000 μl acetonitrile (55% in water, *v/v*) with imatinib-D_3_ (IS-IM-D_3_) as an internal standard (Cayman; Ann Arbor, MI, USA) at 4 °C for 1 h. Ten different concentrations of standard solutions were prepared by sequential dilution in 50% MeOH (*v/v*). For calibration standards, an aliquot of 20 μl for each spiked standard solution was spiked into 200 μl acetonitrile (55% in water, *v/v*) with IS-IM-D_3_. The concentration range for imatinib (Cayman) was 2,000-0 μM and 4-0 μM for carnitine, acetyl carnitine, carnitine D3, and acetyl carnitine D3 (Sigma–Aldrich). Imatinib and carnitine extracts (200 μl) were separated from cell pellets, mixed with 20 μl of 50% MeOH (*v/v*) and centrifuged at 37,000×*g* for 30 min at 4 °C. The prepared samples were then used for LC‒MS/MS quantification.

Metabolites of the TCA cycle, glycolysis and total energy production were extracted from cell pellets by 1000 μl methanol:acetonitrile:water (5:3:2, *v/v/v*). All analytes were extracted at 4 °C for 1 h. To quantify the TCA cycle, glycolysis and total energy production metabolites, cell pellet extracts (500 μl) were dried in a centrifugal vacuum concentrator Savant SPD 131 (Thermo Fisher Scientific) and subsequently dissolved in 100 μl water for quantitative analysis. Detailed sample preparation and concentration ranges for individual TCA cycles, glycolysis and total energy production metabolites are described in detail in the Supplementary Materials.

The extracted analytes were quantified using a Prominence HPLC system (Shimadzu, Kyoto, Japan) coupled with a tandem mass spectrometer (QTRAP 4000; Sciex, Framingham, MA, USA). Analyst v.1.6 from SCIEX was used for the acquisition and analysis of data. The parameters for LC‒MS/MS are listed in detail in the Supplementary Materials.

### 2.4 *In vivo* models

Experiments were performed on NOD. Cg-Prkdcscid Il2rgtm1 Wjl/SzJ (NSG)-immunodeficient female mice (n = 16) aged 8–10 weeks. Treated mice received labelled carnitine Car-D3 (1 mg per mouse, gavage) and VNR (20 mg/kg, intraperitoneally) once per day. The experimental design in the selected group of mice required 24 h of food deprivation to prevent the impact of carnitine intake in an ordinary diet. Thigh muscle were isolated from sacrificed mice. Thigh muscle was quickly frozen at once in liquid nitrogen and powdered by grinding in a prechilled mortar; liquid nitrogen was occasionally added to the mortar to prevent thawing. Once the tissue was ground to a fine powder, it was transferred to a prechilled vial tube. The levels of natural carnitine and Car-D3 were measured in plasma and thigh muscle at 24 h. The levels of metabolites of the TCA cycle and glycolysis and the production of energy-rich molecules were analyzed in thigh muscle. The design of all experiments was approved by the institutional Animal Care and Use Committee of Charles University in Prague.

### 2.5 Statistical analysis

The statistical analyses were performed using Student's t test in MS Excel (Microsoft Corporation, Redmond, WA, USA).

## 3 Results

### 3.1 Imatinib significantly reduced carnitine intake and intracellular concentration, while carnitine did not affect imatinib intake

The *BCR::ABL1-*positive CML cell line KCL-22 and the rhabdomyosarcoma cell line HTB-153 express *SLC22A5* mRNA at comparable levels. The OCTN2 protein level was approximately 2× higher in KCL-22 cells (Fig. S1). To test pharmacodynamic parameters for the cellular absorption of imatinib, HTB-153 cells were cultivated with 8 μM imatinib. This concentration ensured that imatinib was available for cells uniformly as these adherent cells grew in layers. The imatinib concentration for KCL-22 *in vitro* tests was determined to be 0.4 μM–1 μM, which is tolerated for short cultivation up to 96 h [19].

**Figure 1:**
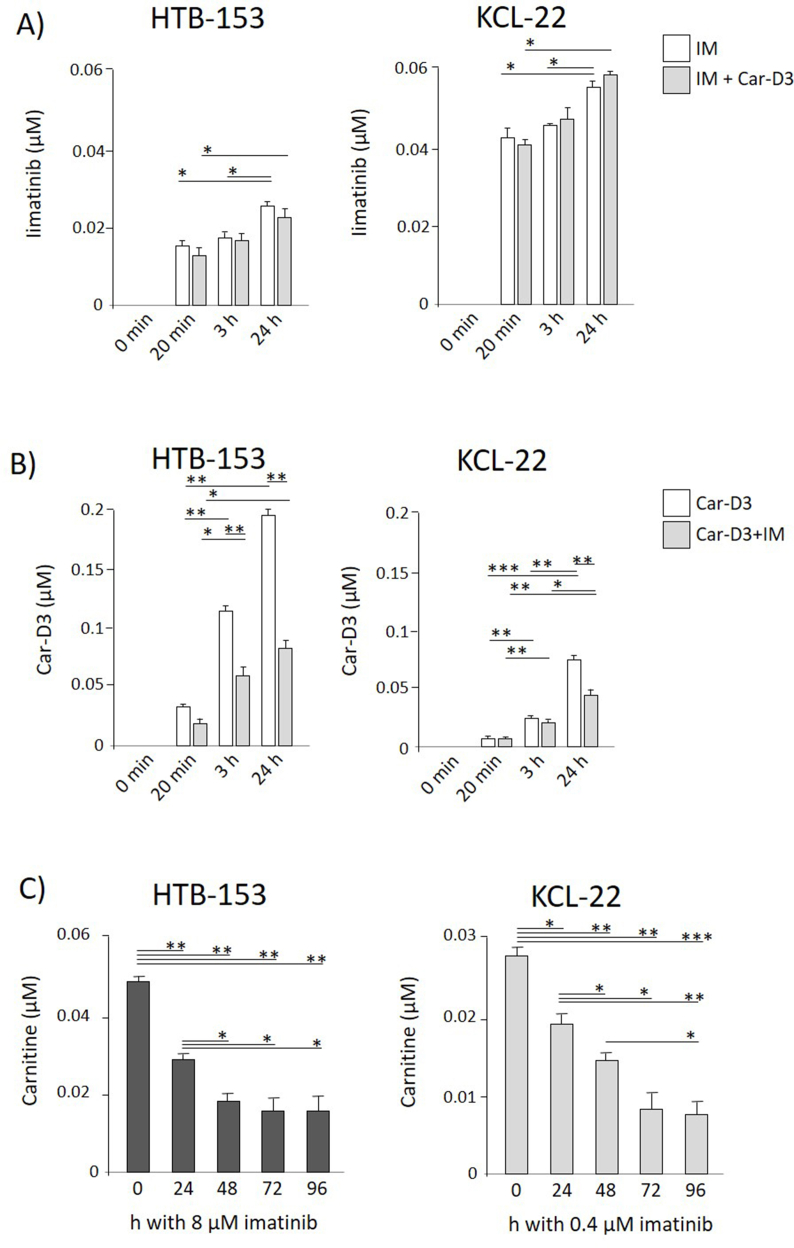
**Carnitine and imatinib intake dynamics.** A) Adherent HTB-153 cells (5 × 10^7^ in 15 ml) and the suspension cell line KCL-22 (10^7^ in 10 ml) were incubated with imatinib (8 μM and 1 μM, respectively) (white bars) or with a combination of imatinib (8 μM and 1 μM, respectively) and labelled carnitine Car-D3 (8 μM and 2.5 μM, respectively) (grey bars). Intracellular levels of imatinib were measured by LC‒MS/MS at 20 min, 3 and 24 h. B) Similarly, HBT-153 and KCL-22 were incubated with Car-D3 alone (white bars) or with the combination of imatinib with carnitine Car-D3 (grey bars). The cells were harvested at 20 min, 3 and 24 h, and the concentration of intracellular Car-D3 was measured. C) Exposure of the cells to imatinib decreased intracellular levels of carnitine. HTB-153 (left graph) and KCL-22 (right) cells were incubated with the indicated doses of imatinib. The level of intracellular natural carnitine was monitored every 24 h up to 96 h of incubation. The cells were collected, and the concentration of intracellular carnitine was measured by LC‒MS/MS. Values are the mean+/−SD (n = 2). Statistics (t test): ∗p ≤ 0.05, ∗∗p ≤ 0.005, ∗∗∗p ≤ 0.0005.

The intracellular concentration of imatinib was measured at 3 time points up to 24 h. The intracellular concentration of imatinib significantly increased after 24 h with the fastest growth during the first 20 min of incubation in cell lines KCL-22 and HTB-153 (Figure 1A, white bars). The concentration values shown in graphs were measured in the extract from cell pellets of the appropriate cell line in the same manner (10 × 10^6^ KCL-22 and 0.5 × 10^6^ HTB-153). The intracellular concentration of isotopically labelled carnitine Car-D3 (which allows us to distinguish labelled carnitine from natural carnitine present in the medium and cells) was measured to study the dynamics of carnitine transport and the hypothesized competition with imatinib intake. The cells were cultivated *in vitro* with 8 μM and 2.5 μM Car-D3 in HTB-153 and KCL-22, respectively. The Car-D3 intracellular concentration was measured at the same time points as imatinib (Figure 1B - white bars). The dynamics of imatinib intake were faster than those of Car-D3. The intracellular concentration of imatinib did not differ between 20 min and 3 h, while the Car-D3 intracellular concentration linearly increased over time, with a 3.4-fold higher concentration at 3 h in HTB-153 cells and a 5-fold higher concentration at 3 h in KCL-22 cells than at 20 min.

Then, we examined whether Car-D3 and/or imatinib intake was influenced if the compounds were added to the cell cultures simultaneously. The intracellular concentration of imatinib in the presence of Car-D3 was comparable to the imatinib intracellular concentration in the absence of Car-D3 in both cell lines (Figure 1A; grey bars). In contrast, the intracellular concentration of Car-D3 in the presence of imatinib was significantly lower in both cell lines compared to the Car-D3 intracellular concentration without imatinib (Figure 1B; grey bars).

Finally, to test the effect of prolonged exposure of the cells to imatinib on intracellular levels of natural carnitine supplied from cultivating medium, the cell lines were incubated with imatinib (8 μM - HTB-153; 0.4 μM - KCL-22) for 96 h. The levels of natural carnitine were continuously checked at time points 0, 24, 48, 72 and 96 h. A significant decrease in the intracellular carnitine concentration was found during first 48 h of incubation in both cell lines with relatively stable lower levels in latter timepoints, presumably reflecting re-established balance between carnitine influx and efflux in the presence of imatinib (Figure 1C).

### 3.2 Carnitine cell intake is renewed after carnitine is added at high concentrations into cell cultures preincubated with imatinib

**Figure 2:**
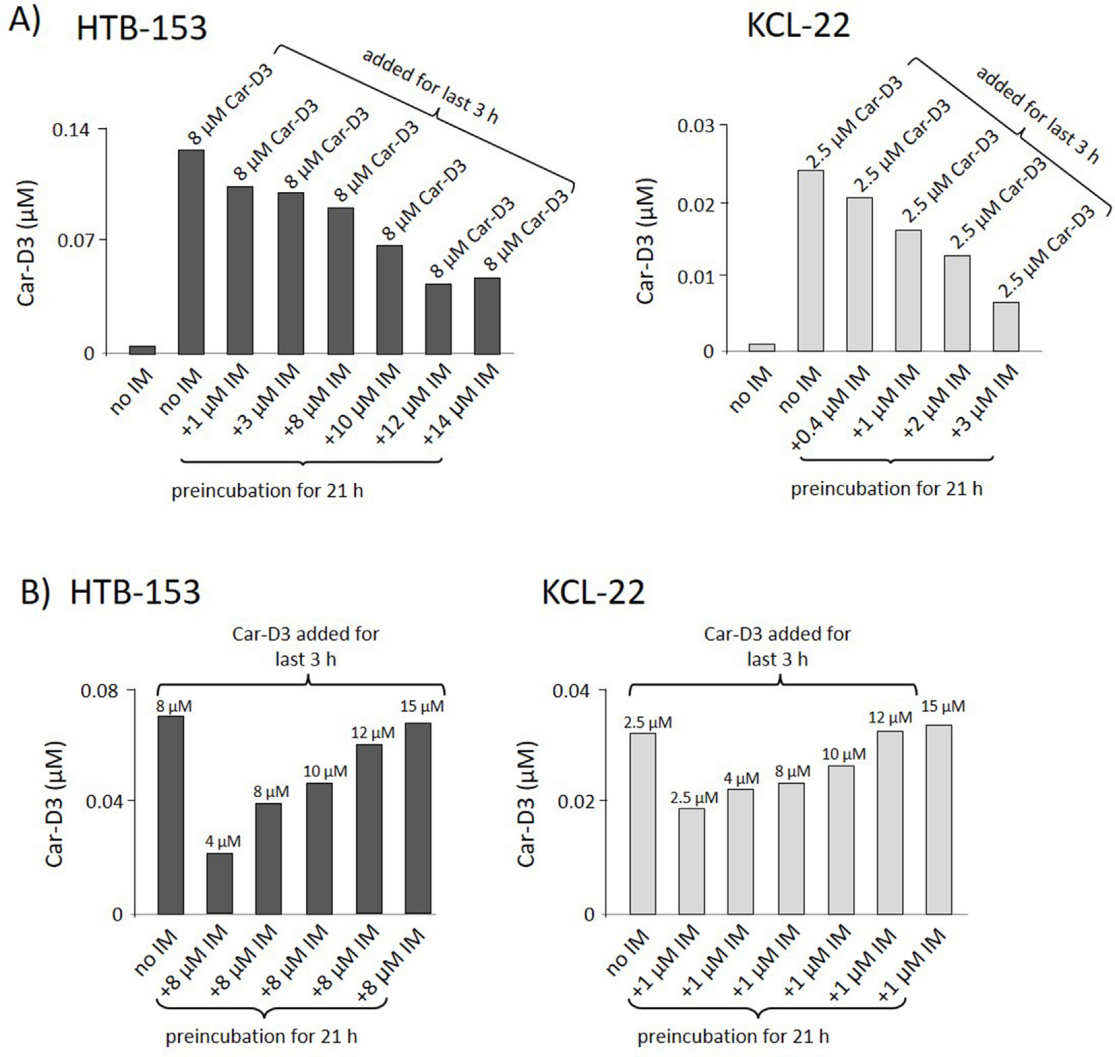
**Preincubation with imatinib reduced the cell intake of carnitine.** A) The cell lines HTB-153 (left graph) and KCL-22 (right) were first preincubated for 21 h with different doses of imatinib (indicated at X-axes). Car-D3 (8 μM for HTB-153 and 2.5 μM for KCL-22) was added for the last 3 h of the 24-hour experiment. The first bar in both graphs represents cultivation without imatinib and Car-D3. B) HTB-153 (left graph) and KCL-22 (right) were first preincubated for 21 h with imatinib (8 μM in the case of HTB-153 and 2.5 μM for KCL-22), and different doses of Car-D3 (dose indicated above bars) were added for the last 3 h of the 24-hour experiment. The first bar in all graphs represents cultivation without imatinib. At the 24-hour time point, the cells were harvested, and the concentration of intracellular imatinib was measured by LC‒MS/MS.

HTB-153 cells were exposed to imatinib for 21 h at different concentrations (ranging from 1 μM to 14 μM). Subsequently, Car-D3 was added to a final concentration of 8 μM for a final 3 h. Intracellular concentrations of Car-D3 decreased with increased concentrations of imatinib during the preincubation phase (Figure 2A). In contrast, the intracellular concentration of imatinib increased in correspondence to the increased level of imatinib added to the culture (Fig. S2A). KCL-22 cells were preincubated for 21 h with different concentrations of imatinib (ranging from 0.4 μM to 3 μM; a tolerable range of imatinib doses for 24 h incubation), and subsequently, Car-D3 was added to a final concentration of 2.5 μM for the final 3 h. Again, while the intracellular concentration of imatinib increased (Fig. S2A), Car-D3 decreased in relation to the level of imatinib used for preincubation (Figure 2A).

Importantly, when a constant concentration of imatinib was used in the preincubation phase (21 h) and Car-D3 was added for the last 3 h at different concentrations (ranging from 4 μM to 15 μM in HTB-153 and from 2.5 μM to 15 μM in KCL-22), the intracellular concentration of Car-D3 corresponded with the increased concentration of added Car-D3 (Figure 2B). In both cell lines, imatinib intake was not influenced by the Car-D3 utilized in the preincubation phase even at the highest dose (Fig. S2B).

To demonstrate this principle, HTB-153 and KCL-22 cells were incubated with the OCTN2-specific inhibitor vinorelbine (VNR), which caused a significant decrease in the intracellular concentration of natural carnitine and intracellular intake of labelled Car-D3 (Figs. S3A and S3B). In contrast, intracellular levels of imatinib that was added to the cell culture at different concentrations for the last 3 h only slightly decreased (Fig. S3C). Data confirmed that OCTN2 is the specific transporter for carnitine but not for imatinib, which is also transported through other transporters.

### 3.3 Subsequent, but not simultaneous, addition of carnitine stimulated energy metabolism attenuated by imatinib in pre-incubated cells

**Figure 3:**
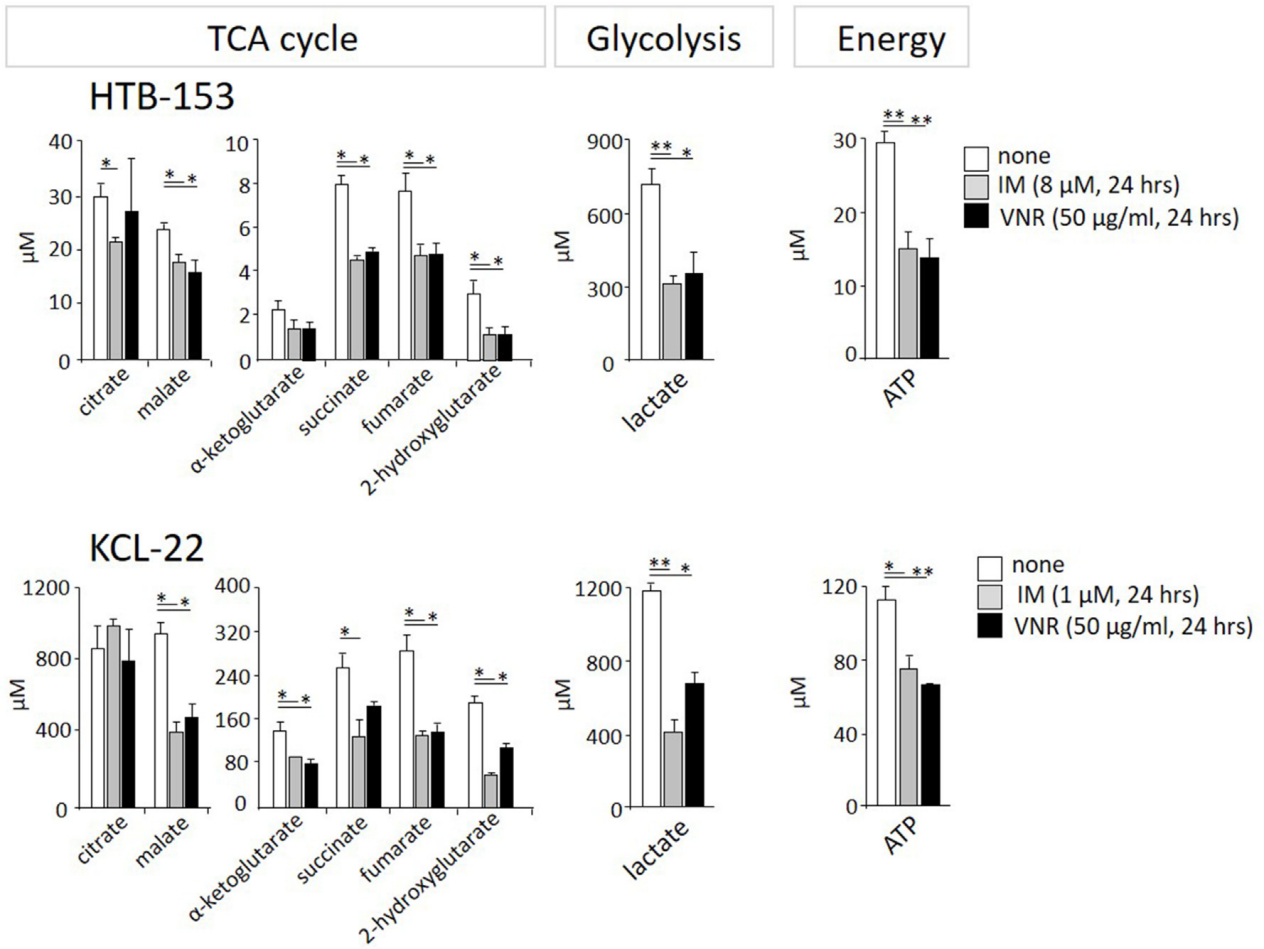
**Energy metabolism pathways suffer from low intracellular carnitine levels after OCTN2 blockade.** HTB-153 (upper row of graphs) and KCL-22 (lower row) cells were incubated for 24 h with either imatinib (grey bars, 8 μM for HTB-153 and 1 μM for KCL-22) or VNR (black bars, 50 μg/ml), and the levels of metabolites of the TCA cycle, glycolysis (lactate) and ATP production were measured by LC‒MS/MS. Untreated cells are depicted as white bars. Values are the mean+/−SD (n = 2). Statistics (t test): ∗p ≤ 0.05, ∗∗p ≤ 0.005, ∗∗∗p ≤ 0.0005.

Both cell lines were incubated for 24 h with imatinib (8 μM in HTB-153; 1 μM in KCL-22) or VNR (50 μg/ml in both cell lines). The concentration of the TCA metabolites (citrate, malate, succinate, fumarate, 2-hydroxyglutarate) significantly decreased compared to that of untreated cells. Additionally, lactate (a product of glycolysis) and total ATP were significantly decreased in HTB-153 cells (Figure 3). The same results were observed in KCL-22 except for citrate (no change in the level) and α-ketoglutarate (significantly decreased level).

**Figure 4:**
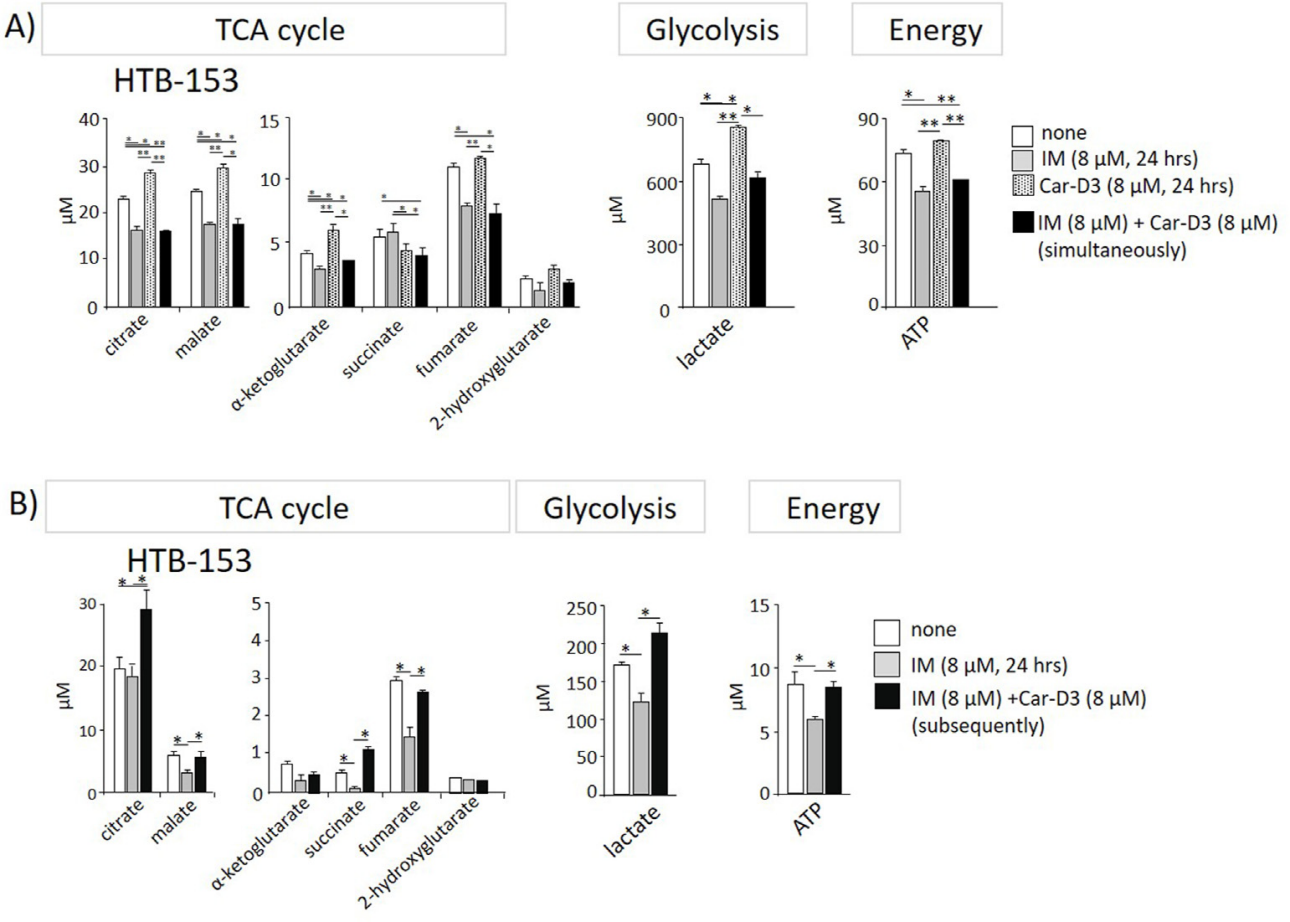
**The addition of carnitine stimulates energy metabolism, which is first attenuated after imatinib treatment.** A) HTB-153 cells were incubated for 24 h with 8 μM imatinib (grey bars), 8 μM carnitine Car-D3 (dotted bars) or both chemicals simultaneously (black bars). Untreated cells are depicted as white bars. B) HTB-153 cells were incubated for 24 h either with 8 μM imatinib (grey bars) or pre-incubated with 8 μM imatinib for 21 h, and 8 μM Car-D3 was added for the last 3 h of the 24-hour experiment (black bars). Untreated cells - white bars. The levels of metabolites of the TCA cycle and glycolysis and the production of ATP were measured by LC‒MS/MS. Values are the mean+/−SD (n = 2). Statistics (t test): ∗p ≤ 0.05, ∗∗p ≤ 0.005, ∗∗∗p ≤ 0.0005.

Then, we tested whether Car-D3 addition to both cell lines influences the levels of the studied metabolites and compared the results with the simultaneous administration of Car-D3 and imatinib. Except for succinate, fumarate, 2-hydroxyglutarate and ATP, a significant increase in the metabolites was observed when Car-D3 was added to the culture alone, confirming its role in energy metabolism (Figure 4A; dotted bars). From previous results, we learned that imatinib intake occurred more quickly and outcompete carnitine as substrate for OCTN2, resulting in a significant reduction in Car-D3 intracellular levels. Consequently, simultaneous addition of Car-D3 with imatinib led to significant decreases in citrate, malate, α-ketoglutarate, fumarate, and lactate in HTB-153 cells (Figure 4A; black bars). The same effect was observed after incubation with imatinib alone because imatinib also blocked natural carnitine intake from cultivating medium (Figure 4A; grey bars). Corresponding data were observed in the KCL-22 cell line (Fig. S4).

From previous data, we knew that adding Car-D3 to a culture preincubated with imatinib renewed Car-D3 intake. Therefore, we examined effect of Car-D3 on metabolite levels when HTB-153 was incubated for 21 h with imatinib, and for the last 3 h, Car-D3 was added. In agreement with renewed carnitine intake, the subsequent addition of Car-D3 rescued energy metabolism impaired by imatinib and the levels of citrate, malate, succinate, fumarate, lactate and ATP significantly increased (Figure 4B).

### 3.4 OCTN2 inhibition by vinorelbine *in vivo* decreased the rate of the Krebs cycle and production of ATP in thigh tissue

Mouse models were used to test whether the observed impact of reduced intracellular carnitine levels on cell metabolism *in vitro* can be achieved *in vivo*. VNR was used as the OCTN2-specific inhibitor that was applied to mice intraperitoneally. Imatinib, which needs to be applied through gavage, was not used due to its extremely fast pharmacodynamics; the pharmacotherapeutic window available is narrower in mice than humans. Therefore, to determine the effect of imatinib on the carnitine cycle and metabolism in mice, several tests would be needed to measure the frequency of imatinib administration through gavage. Therefore, in line with the ethical guidelines for animal use in research, we did not perform tests to determine the imatinib dose and frequency of feeding because tens of mice would be needed.

Five groups of mice in the 24-hr experiment were i) untreated, ii) food deprived since the start of experiment, iii) food deprived and fed with labelled carnitine only (Car-D3, 1 mg per mice, gavage), iv) food deprived and treated with VNR (20 mg/kg, intraperitoneally) and v) food deprived fed with Car-D3 only and treated with VNR. Food deprivation was important for preventing the impact of carnitine intake in an ordinary diet. The levels of natural carnitine and Car-D3 were measured in plasma (Fig. S5a) and thigh muscle (Fig. S5B) at 24 h. Food deprivation led to significantly decreased total levels of carnitines (Car and Car-D3). However, no difference was found in the levels measured in thigh tissue.

**Figure 5:**
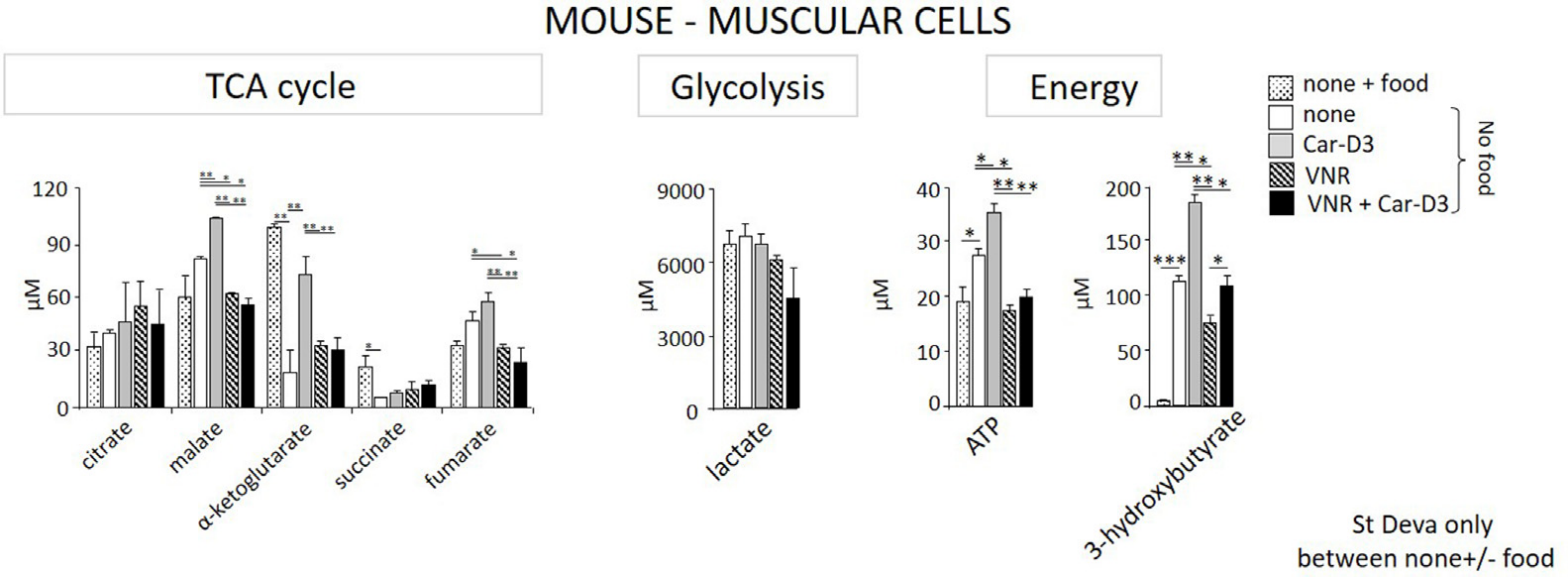
**Blocking carnitine cell uptake by muscular cells downregulates the rate of TCA and the production of ATP *in vivo*.** Five groups of mice (two mice) were either untreated or untreated and food deprived or food deprived and treated with carnitine Car-D3 (dose 1 mg per mouse, gavage), VNR (20 mg/kg, intraperitoneally) or a combination of VNR and Car-D3. The impact of food deprivation (white bars) of food deprivation with treatment (grey- Car-D3, dotted- VNR and black bars- VNR + Car-D3) on the levels of metabolites of the TCA cycle, glycolysis and production of ATP and 3-hydroxybutyrate was measured by LC‒MS/MS in thigh muscular tissue at 24 h. Values are the mean+/−SD (n = 2). Statistics (t test): ∗p ≤ 0.05, ∗∗p ≤ 0.005, ∗∗∗p ≤ 0.0005.

The levels of metabolites of the TCA cycle and glycolysis and the production of energy-rich molecules were analyzed in thigh tissue. Significantly increased concentrations of the ketone body 3-hydroxybutyrate were found in the thigh tissue of all groups of food-deprived mice, which corresponded to the fasting state (Figure 5). Concerning TCA metabolites, a decrease in α-ketoglutarate and succinate levels was observed in food-deprived mice compared to the control group. However, in mice fed Car-D3, the production of α-ketoglutarate, malate, fumarate and ATP was significantly stimulated compared to that of food-deprived mice or food-deprived mice treated with the OCTN2 blocker VNR. The impact of OCTN2 inhibition by VNR on Car-D3-fed mice was observed in significantly reduced production of α-ketoglutarate, malate, fumarate, ATP and 3-hydroxybutyrate (Figure 5). The concentration values of all chemicals and metabolites were measured after extraction from 60 μg of homogenized thigh muscle.

## 4 Discussion

Muscle cramps and fatigue are frequently reported side effects of patients with CML treated with imatinib [2,3], which significantly influence patient QoL [4]. Carnitine acts as the transporter of long-chain fatty acids into the mitochondria for oxidation. This leads to the production of an acetyl-CoA pool that enters the TCA cycle and produces energy, which is essential for releasing the actin–myosin bond [20,21]. In a recent work, the toxicity of TKIs (with the majority of imatinib-treated patients) on skeletal muscle was studied, demonstrating that CML patients with muscular complaints showed delayed quadriceps femoris muscle force generation and a trend towards delayed relaxation in fatigued muscle compared to patients without muscle adverse events [22]. Molecular mechanisms describing these imatinib side effects wait to be uncovered. Our work demonstrates that imatinib treatment impacts carnitine intake, resulting in a significantly decreased intracellular concentration, which affects the metabolism of long-chain fatty acids and TCA cycle. We focused on measuring changes in the TCA cycle, glycolysis, and overall production of ATP *in vitro* and *in vivo*.

*BCR::ABL1*-positive cells express the high-affinity glucose transporter GLUT-1 and consequently increase glucose uptake and glycolysis [23,24]. After exposure of *BCR::ABL1*-positive cells to imatinib, the level of glycolytic enzymes and lactate production decreased, while the activity of the TCA cycle and oxidative phosphorylation increased, indicating the Warburg effect [[25], [26], [27]]. The application of a higher dose of imatinib, however, affected the TCA cycle and diminished ATP production [25]. Other works brought similar observations using muscle cell lines. Exposure to high concentrations of imatinib led to the inhibition of oxidative phosphorylation, decreased ATP production and decreased mitochondrial copy numbers in C2C12 murine myoblasts, C2C12 murine myotubes and human rhabdomyosarcoma RD cells [28,29]. In line with these results, our work showed a significant reduction in lactate production and the rate of the TCA cycle after exposure of the *BCR::ABL1*-positive cell line KCL-22 and rhabdomyosarcoma HTB-153 cells to high concentrations of imatinib accompanied by significant ATP depletion. This mitochondrial metabolism impairment is a consequence of imatinib transportation through the OCTN2 carnitine-specific transporter that caused decreased carnitine intracellular concentration in both cell lines. In addition, OCNT2 inhibition by vinorelbine in food-deprived mice and in mice fed carnitine only, resulted in a decreased rate of the TCA cycle and decreased production of ATP in thigh tissue. Other possible, mutually not exclusive mechanisms of mitochondrial toxicity in muscle cells after exposure to imatinib include inhibition of Complex I and increased production of reactive oxygen species [28] or inhibition of Complex V and decreased nucleoside uptake [29].

Recently, a promising clinical trial was started concerning the role of carnitine consumption in relieving cramps in patients with advanced gastrointestinal stromal tumors treated with imatinib [30]. The first published data showed that 500 mg carnitine oral supplementation two or three times daily was safe and effective in improving indicators of QoL, such as basic activities of daily living, sleeping, and outdoor activity with no reported adverse effect of carnitine. These observations were in line with previous studies with a positive impact of carnitine supplementation in hemodialyzed patients (low serum carnitine due to poor nutrition, deprivation of endogenous synthesis from kidneys, and removal by hemodialysis), which ameliorated muscle cramps [31]. The clinical data are supported by the results of our work showing that carnitine consumption in food-deprived mice led to the significantly increased production of metabolites of the TCA cycle (malate, fumarate, α-ketoglutarate) and ATP in thigh muscle. Moreover, the addition of carnitine to the culture of HTB-153 muscle cells pre-treated with imatinib significantly increased the levels of citrate, malate, succinate, and fumarate of the TCA cycle and the production of ATP. This was not observed when carnitine and imatinib were simultaneously added reflecting non-equal competition for OCTN2. In the case of carnitine supplementation, the time of administration after imatinib consumption seems to be important and will require further testing. According to pharmacokinetics and pharmacodynamics data, the peak of imatinib plasma concentration is 5 h after imatinib consumption, which decreases over time, with the lowest peak after the next 5 h [32]. Thus, carnitine administration, e.g., 10 h (±2 h) after imatinib consumption, may ensure effective carnitine transportation to cells. Importantly, even simultaneous addition of carnitine and imatinib did not influence imatinib intake into CML cells. This is especially relevant from a clinical point of view, indicating that carnitine supplementation does not affect/prevent imatinib influx into leukemic cells to block BCR::ABL1 kinase activity.

In conclusion, this preclinical experimental work showed that intracellular concentrations of carnitine decrease in cells due to preferential intake of imatinib through the carnitine-specific transporter OCTN2. The decreased carnitine intake in muscular cells impaired the Krebs cycle and ATP production. This mechanism of action may be related to side effects, such as fatigue, muscle cramps and musculoskeletal pain that are frequently reported in CML patients (and patients with advanced gastrointestinal stromal tumors) treated with imatinib and to much lesser degree also with other TKIs. Carnitine supplementation after imatinib treatment *in vitro* and *in vivo* led to increased carnitine intake and renewal of mitochondrial energy production. Following suggestive results of above-mentioned trial regarding imatinib-treated gastrointestinal tumors, our data provide a good rationale to support a clinical investigation of using carnitine as a dietary supplement in CML patients suffering from fatigue and muscular side effects.

## CRediT authorship contribution statement

**Pavel Burda:** Writing – original draft, Validation, Methodology, Investigation, Data curation, Conceptualization. **Alzbeta Hlavackova:** Validation, Methodology, Formal analysis. **Vendula Polivkova:** Project administration, Methodology, Formal analysis. **Nikola Curik:** Writing – original draft, Project administration, Investigation, Data curation. **Adam Laznicka:** Investigation. **Jitka Krizkova:** Methodology, Formal analysis. **Jiri Suttnar:** Methodology, Formal analysis. **Pavel Klener:** Methodology, Formal analysis. **Katerina Machova Polakova:** Writing – original draft, Supervision, Funding acquisition, Conceptualization.

## Declaration of competing interest

The authors declare that they have no known competing financial interests or personal relationships that could have appeared to influence the work reported in this paper.

## Data Availability

Data will be made available on request.
