# Supplementary material for: Imatinib therapy of chronic myeloid leukemia significantly reduces carnitine cell intake, resulting in adverse events
Source: Mol Metab. 2024 Aug 23;88:102016. doi: 10.1016/j.molmet.2024.102016 (PMC11403060; doi:10.1016/j.molmet.2024.102016)
Supplement: Multimedia component 1 [file mmc1.docx]

**Supplemental Figures**


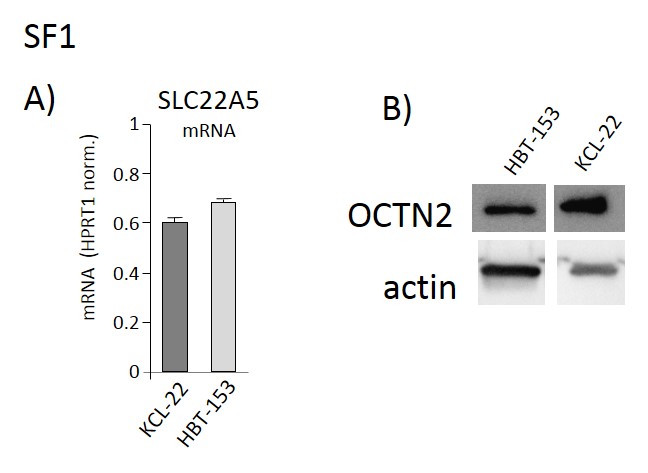


**SF1** - OCTN2 gene expression in KCL-22 and HTB-153 cells. A) Y-axis: 2exp (-ΔCT) expression relative to HPRT1 expression. Values are the mean+/-SD (n=3). B) Protein level analysis of OCTN2. Actin expression used as housekeeping control.


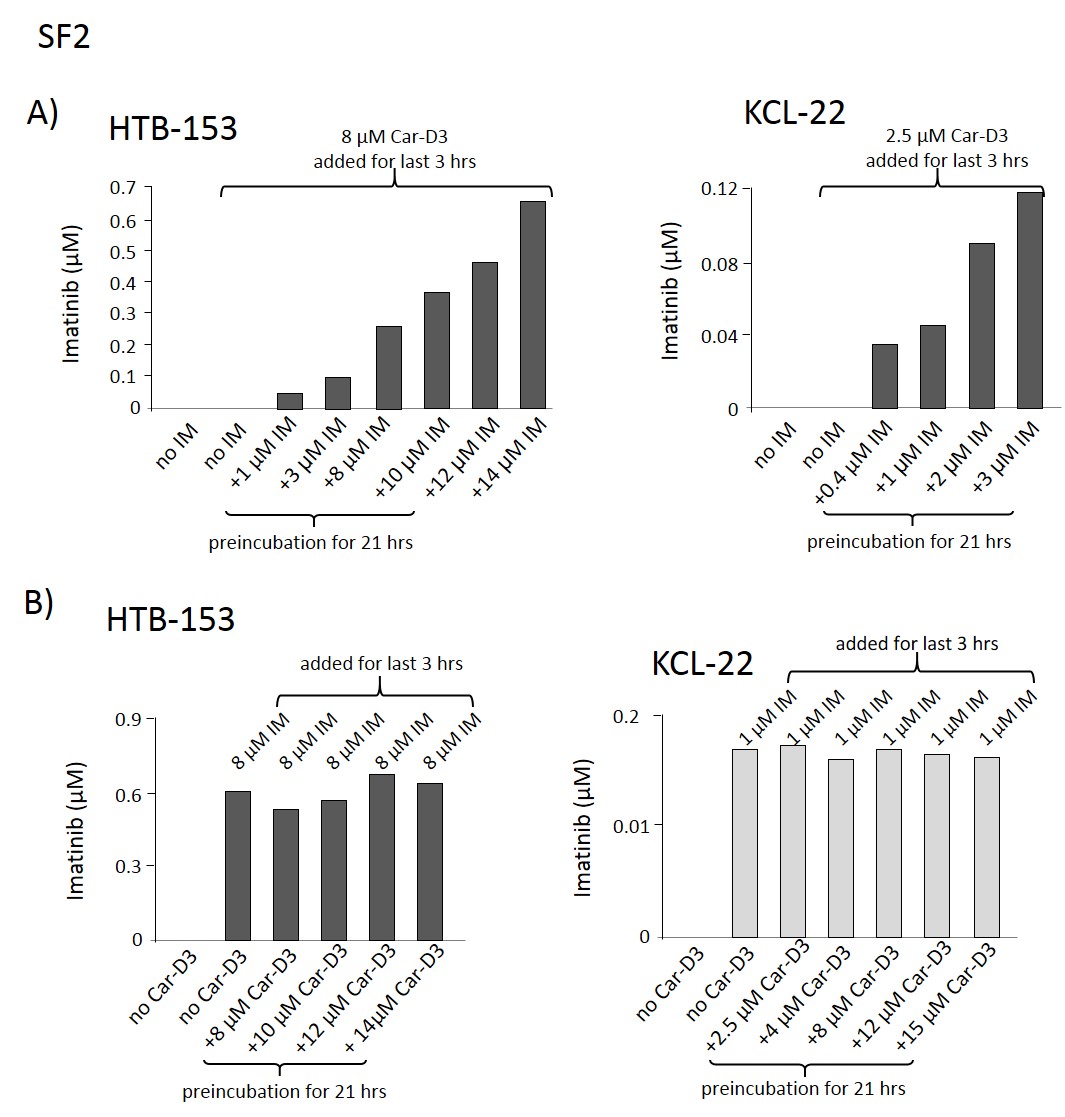


**SF2 -** A) Cell lines HTB-153 (left graph) and KCL-22 (right) were firstly preincubated for 21 hours with different doses of imatinib (indicated at X-axes), Car-D3 (8 µM in case of HTB-153 and 2.5 µM for KCL-22) was added for last 3 hours of 24 hours. experiment. At 24-hour time point the cells were harvested and concentration of intracellular imatinib measured by LC-MS/MS. B) HTB-153 (left graph) and KCL-22 (right) were firstly preincubated for 21 hours with different doses of Car-D3 (indicated at X-axis), imatinib (8 µM for HTB-153 and 1 µM in case of KCL-22) was added for last 3 hours of 24 hours experiment. At 24 hours the cells were harvested and intracellular imatinib measured by LC-MS/MS.


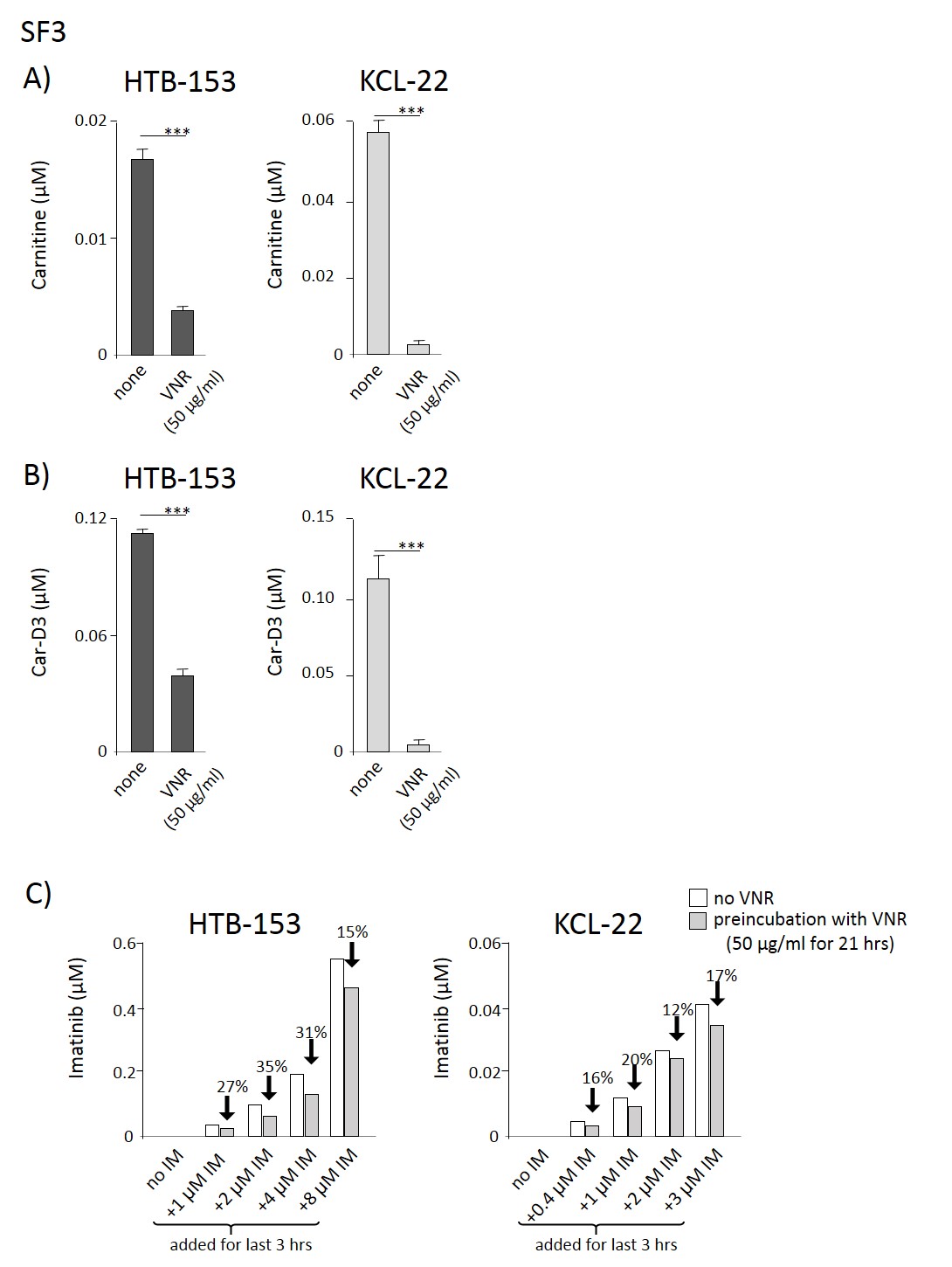


**SF3 -** Cell lines HTB-153 (left graph) and KCL-22 (right) were incubated with vinorelbine (VNR) (concentration 50 µg/ml) and levels of intracellular carnitine (A) was measured by LC-MS/MS at 24 hrs. B) The levels of Car-D3 was measured in cells firstly pretreated for 21 hours with VNR (50 µg/ml) and subsequently exposed to Car-D3 for 3 hours (right bars, 8 µM Car-D3 in case of HTB-153 and 2.5 µM for KCL-22) ). Left bars show Car-D3 concentration in cells after simple incubation with Car-D3 for 3hrs. C) HTB-153 (left graph) and KCL-22 (right) were firstly untreated (white bars) or pre-treated for 21 hours with VNR (50 µg/ml, gray bars). Subsequently, different doses of imatinib (indicated at X-axes) were added for 3 hrs. Cells were harvested and concentration of intracellular imatinib measured by LC-MS/MS. The measure of decrease relative (in %) to cell culture not pre-treated with VNR depicted above gray bars.


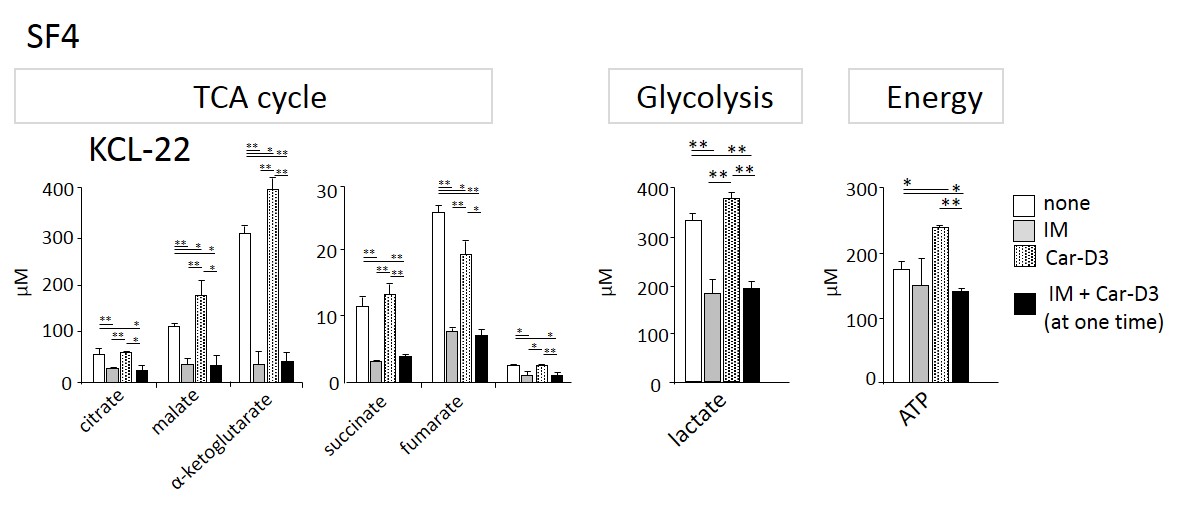


**SF4 -** Simultaneous addition of carnitine with imatinib induce similar affect as addition of imatinib alone. Cell line KCL-22 was incubated for 24 hours either with 1 µM imatinib (gray bars) or 2.5 µM carnitine Car-D3 (dotted bars) or both chemicals together at the same time (black bars). Untreated cells are depicted as white bars. The levels of metabolites of TCA cycle, glycolysis and production of ATP were measured by LC-MS/MS. Values are the mean+/-SD (n=2). Statistics (t-test): * p ≤0.05, ** p ≤0.005, *** p ≤0.0005.


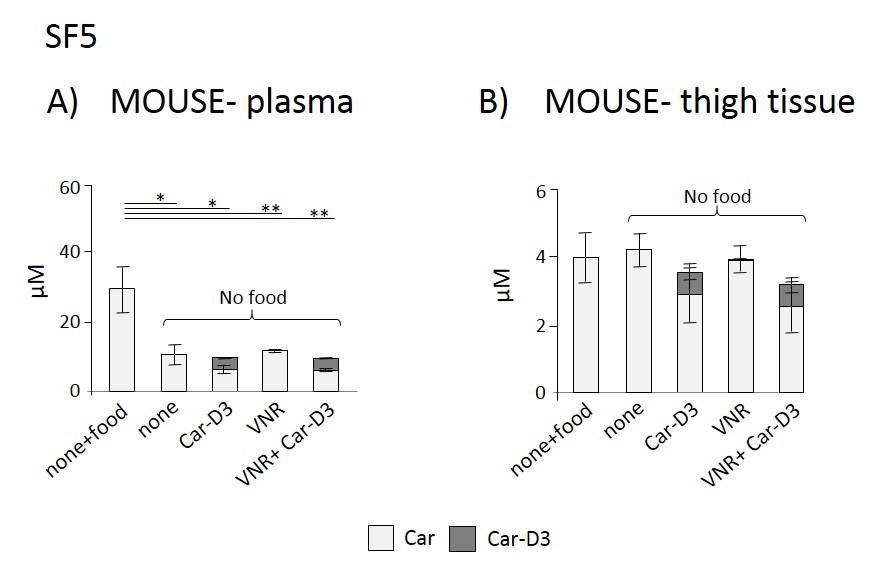


**SF5 -** Five group of mice (à two mice) were either untreated or untreated and food deprived or food deprived and treated by carnitine Car-D3 (dose 1mg per mice, gavage), VNR (20 mg/kg, intraperitoneally) or with combination of VNR and Car-D3. The level of natural carnitine and labelled Car-D3 were measured in plasma (A) and muscle cell (B) at the time 24 hrs.
