# Supplementary material for: Imatinib therapy of chronic myeloid leukemia significantly reduces carnitine cell intake, resulting in adverse events
Source: Mol Metab. 2024 Aug 23;88:102016. doi: 10.1016/j.molmet.2024.102016 (PMC11403060; doi:10.1016/j.molmet.2024.102016)
Supplement: Multimedia component 2 [file mmc2.docx]

**[Supplementary Materials](https://www.ncbi.nlm.nih.gov/pmc/articles/PMC5364945/)**

**Measurement of intracellular concentration of imatinib, carnitine and TCA cycle, glycolysis and total energy production metabolites**

**LC-MS/MS Parameters for imatinib** q**uantification**

Samples (5 ul) were injected onto Atlantis dC18 column (50x2.1 mm, 5 µm; Waters, Prague, Czech Republic). The analytic column was kept at 45 °C. The mobile phases were composed of 0.05% ammonium acetate/5% methanol (*v/v*) (A) and 0.05% ammonium acetate/methanol (*v/v*) (B). The flow rate was 0.25 ml/min. Chromatographic separation of the analytes was performed using a linear gradient as follows: t (min)/%B: 0/40, 3/100, 6/100, 6.5/40. Total run time was 9 min. Imatinib and IS-IM-D3 were detected by tandem mass spectrometry working in positive multiple reaction monitoring (MRM) mode. The ion source was operated using ion spray voltage set at 5000 V, curtain gas at 30 psi, ion source temperature at 550 °C, ion source gas 1 at 40 psi, ion source gas 2 at 60 psi, and collision gas at medium. Imatinib and IS-IM-D3 was monitored at *m/z* 494.3→394.1 and 497.3→394.2 respectively, declustering potential voltage (DP), 116 V; collision energy (CE), 37 V; collision cell exit potential (CXP), 28 V, and entrance potential (EP), 10 V.

**LC-MS/MS Parameters for carnitines** q**uantification**

Carnitines were separated using XBridge Amide column (50x2.1 mm, 3.5 µm; Waters, Milford (MA), USA). Sample injection volume was 1 ul. The analytic column was kept at 35 °C. The mobile phases were composed of 5mM ammonium formate (pH=3.2)/90% acetonitrile (*v/v*) (A) and 5mM ammonium formate (pH=3.2) (B). The flow rate was 0.3 ml/min. Chromatographic separation of the analytes was performed using a linear gradient as follows: t (min)/%B: 0/5, 2/10, 4/80, 5/80, 6/5. Total run time was 12 min. Carnitines were detected by tandem mass spectrometry working in positive MRM mode. The ion source was operated using ion spray voltage set at 5500 V, curtain gas at 30 psi, ion source temperature at 500 °C, ion source gas 1 at 45 psi, ion source gas 2 at 45 psi, and collision gas at medium. Carnitines were monitored at parameters listed in Table X.

Table X. QTRAP 4000 mass spectrometer settings for carnitines.

| **Analyte** | **Q1 m/z** | **Q3 m/z** | **DP [V]** | **CE [V]** | **CXP [V]** |
| --- | --- | --- | --- | --- | --- |
| AC-d3 | 207.1 | 84.9 | 41 | 29 | 16 |
| AC | 204.1 | 84.9 | 61 | 29 | 16 |
| C-d3 | 165.2 | 84.9 | 66 | 29 | 16 |
| C | 162.2 | 84.8 | 46 | 29 | 16 |
| IS-IM-d3 | 497.3 | 394.2 | 116 | 37 | 28 |

Q1- declustering potential voltage (DP); collision energy (CE); collision cell exit potential (CXP); entrance potential (EP); the mass-to-charge ratio of precursor ion in the first quadrupole (Q1 m/z); the mass-to-charge ratio of product ion in the third quadrupole (Q3 m/z); C-carnitine ; AC- acetylcarnitine; C-d3 - labelled carnitine; AC-d3 – labelled acetylcarnitine.

**Quantitative determination of TCA cycle, glycolysis and total energy production metabolites**

TCA cycle, glycolysis and total energy production metabolites were analyzed by LC-MS/MS according Koralkova et al. 2021. ^1^

Briefly, after extraction and dissolving of dried supernatant in 100 µl water, samples were mixed with 20 µl of internal standards mixture. Nine different concentrations of standards solution were prepared by sequential dilution in water. For calibration standards, an aliquot of 50 µl for each spiking standard solution was mixed with 10 µl acetonitrile internal standards mixture. Final concentrations of internal standards and concentration ranges of individual metabolites are listed in Table XY. Samples and calibration standards were centrifuged at 37,000x g for 30 min at 4 °C. The samples thus prepared were used for LC-MS/MS quantification.

Concentration ranges of individual metabolites are listed in Table XY:

| **Metabolite** | **Concentration range [µM]** |
| --- | --- |
| Citrate | 0-250 |
| Malate | 0-50 |
| α-ketoglutarate | 0-50 |
| Succinate | 0-50 |
| Fumarate | 0-50 |
| 2-hydroxyglutarate | 0-50 |
| Lactate | 0-6000 |
| ATP | 0-50 |
| 3-hydroxybutyrate | 0-500 |
| **Internal standard** | **Final concentration [µM]** |
| Citrate 13C6 | 200 |
| Lactate 13C3 | 2500 |
| Fumarate 13C4 | 45 |
| Succinate 13C4 | 50 |
| Malate D3 | 50 |
| α-ketoglutarate 13C4 | 50 |
| ATP 13C10 | 25 |
| D-2-hydroxyglutarate 13C5 | 27 |

TCA cycle, glycolysis and total energy production metabolites were separated using ZIC-pHILIC column (50 × 2.1 mm, 5 μm) (Sigma-Aldrich, Prague, Czech Republic). The injection volume was 1 µl. The column was kept at 35°C. The mobile phases were composed of 20 mM ammonium acetate, pH 9.0/5% acetonitrile (*v/v*) with medronic acid (5 μM, final concentration; Sigma-Aldrich, Prague, Czech Republic) (A) and 20 mM ammonium acetate, pH 9.0/90% acetonitrile (*v/v*) with medronic acid (5 μM, final concentration) (B). The flow rate was 0.25 ml/min. Chromatographic separation of the analytes was performed using a linear gradient as follows: t (min)/% B: 0/100, 6/20, 7.5/20, 8/100. Total run time was 15 min. TCA cycle, glycolysis and total energy production metabolites were detected by tandem mass spectrometry working in negative MRM mode. The ion source was operated using ion spray voltage set at 4500 V, curtain gas at 30 psi, ion source temperature at 475 °C, ion source gas 1 at 33 psi, ion source gas 2 at 33 psi, and collision gas at medium. TCA cycle, glycolysis and total energy production metabolites were monitored at parameters listed in Table Y.

Table Y. QTRAP 4000 mass spectrometer settings for TCA cycle, glycolysis and total energy production metabolites.

| **Metabolite** | **Q1 *m/z*** | **Q3 *m/z*** | **DP [V]** | **CE [V]** | **CXP [V]** |
| --- | --- | --- | --- | --- | --- |
| Citrate | 190.8 | 110.7 | -45 | -18 | -7 |
| Malate | 132.8 | 114.7 | -35 | -16 | -5 |
| α-ketoglutarate | 145.0 | 100.8 | -30 | -12 | -5 |
| Succinate | 116.8 | 73,0 | -45 | -18 | -5 |
| Fumarate | 114.8 | 70.8 | -35 | -12 | -1 |
| 2-hydroxyglutarate | 146.8 | 128.8 | -40 | -14 | -11 |
| Lactate | 88.9 | 42.8 | -45 | -20 | -5 |
| ATP | 505.9 | 78.9 | -70 | -116 | -13 |
| 3-hydroxybutyrate | 102.9 | 58.9 | -35 | -16 | -9 |
| **Internal standard** | **Q1 *m/z*** | **Q3 *m/z*** | **DP [V]** | **CE [V]** | **CXP [V]** |
| Citrate 13C6 | 196.8 | 115.7 | -45 | -18 | -5 |
| Lactate 13C3 | 91.8 | 44.9 | -20 | -24 | -1 |
| Fumarate 13C4 | 118.8 | 73.8 | -40 | -12 | -1 |
| Succinate 13C4 | 120.8 | 75.8 | -40 | -16 | -1 |
| Malate D3 | 135.8 | 116.8 | -40 | -18 | -5 |
| α-ketoglutarate 13C4 | 148.8 | 104.9 | -30 | -12 | -5 |
| ATP 13C10 | 516.1 | 78.8 | -70 | -96 | -13 |
| D-2-hydroxyglutarate 13C5 | 368.1 | 151.9 | -35 | -12 | -9 |

Q1- declustering potential voltage (DP); collision energy (CE); collision cell exit potential (CXP); entrance potential (EP); the mass-to-charge ratio of precursor ion in the first quadrupole (Q1 m/z); the mass-to-charge ratio of product ion in the third quadrupole (Q3 m/z).

Analyst v.1.6 from Sciex was used for all the acquisition and analysis of data.

**Reference**

1. Koralkova P, Belickova M, Kundrat D, et al. Low Plasma Citrate Levels and Specific Transcriptional Signatures Associated with Quiescence of CD34(+) Progenitors Predict Azacitidine Therapy Failure in MDS/AML Patients. *Cancers (Basel)*. 2021;13(9).
